# An Account of Diseases in an Eastern District of London, from Jan. 20, to Feb. 20, 1811

**Published:** 1811-03

**Authors:** 


### *An Account of Diseases in an Eastern District of London, from Jan. 20, to Feb. 20, 1811.*

| | |
|---|---|
| **ACUTE DISEASES.** | |
| Pleuritis | 2 |
| Pneumonia | 1 |
| Peripneumonia Notha | 4 |
| Icterus | 1 |
| Rheumatismus Acutus | 5 |
| **CHRONIC DISEASES.** | |
| Tussis | 20 |
| Dyspnœa | 12 |
| Tussis cum Dyspnœa | 17 |
| Catarrhus | 4 |
| Raucedo | 3 |
| Phthisis Pulmonalis | 4 |
| Hœmoptysis | 3 |
| Cephalalgia | 4 |
| Vertigo | 2 |
| Ophthalmia | 2 |
| Epistaxis | 1 |
| Amenorrhœa | 4 |
| Enterodynia | 3 |
| Obstipatio | 4 |
| Hœmorrhois | 2 |
| Rheumatismus Chronicus | 18 |

| PUERPERAL DISEASES. | | INFANTILE DISEASES. | |
|---|---|---|---|
| Ephemera - - | 5 | Ophthalmia - | 3 |
| Menorrhagia Lochialis | 4 | Erysipelas Infantile | 2 |
| Dolor post partum | 7 | Febris Mesenterica | 3 |
| Peritonitis - | 2 | Aphthæ - - | 4 |
| | | Herpes - - | 6 |

As by similar causes similar effects will be produced, so it may well be expected that a uniformity will appear in the accounts which are given of the diseases by which the present season is distinguished. During the winter months, and in the earlier part of the vernal quarter, diseases of the chest form a prominent feature in the list. Coughs, catarrhs, inflammation of the lungs, hæmoptysis, and other affections of the pulmonary system, engage a very large proportion of medical attention. In some subjects, and under some peculiar circumstances, these complaints assume a very formidable aspect. To those who are labouring under the weight of years, and especially to those who have been accustomed to annual returns of one, or other of these maladies, every recurrence of them must be productive of considerable anxiety. In several of the instances referred to in the list a fatal termination took place. In others where there was a more favourable issue, there is reason to suspect that though the patient escaped from the worst effects of primary disease, he may fall a sacrifice to others which very frequently succeed it. Hydrops pectoris too often occurs after some severe attacks of peneumonic disease. This complaint approaches in so insidious a manner that its presence is hardly suspected. Though under the influence of this disease the respiratory functions are impeded, yet this effusion appears trifling, in comparison of what has been experienced under the acute state of the previous disease, and the patient considers himself as convalescent, rather than labouring under a new disease. The anasarcous swellings, however, which soon appear, paucity of urine, palpitation of the heart, sudden sense of suffocation upon first awaking in the night, and other symptoms, soon characterize the disease. Into this state, several of the patients referred to in the list seem to be in danger of falling.

